# Design and Performance Analysis of Spiral Microchannels for Efficient Particle Separation Using Inertial Microfluidics

**DOI:** 10.3390/mi16030349

**Published:** 2025-03-19

**Authors:** Eda Ozyilmaz, Gamze Gediz Ilis

**Affiliations:** 1 Department of Mechanical Engineering, Gebze Technical University, 41400 Gebze, Kocaeli, Türkiye; ggediz@gtu.edu.tr; 2 Division of Engineering in Medicine, Department of Medicine, Brigham and Women’s Hospital, Harvard Medical School, Cambridge, MA 02139, USA

**Keywords:** microfluidic device, particle separation, inertial microfluidics, spiral microchannels, performance analysis, Taguchi method, design optimization

## Abstract

Accurate separation in microfluidic devices is crucial for biomedical applications; however, enhancing their performance remains challenging due to computational and experimental constraints. This study aims to optimize microfluidic devices by systematically refining spiral microchannel configurations for the segregation of circulating tumor cells (CTCs) and red blood cells (RBCs) through detailed variable analysis and resource-efficient techniques. The spiral design was developed into six variations, considering loop numbers (2, 3, and 4), aspect ratios (2.333, 3.333, and 5), spiral radii (5, 6, and 7 mm), flow rates (1.5, 2, and 3 mL/min), surface roughness levels (0, 0.5, and 1 μm), and particle sizes (12, 18, and 24 μm). Simulations were conducted in COMSOL Multiphysics and evaluated using the Taguchi method to determine the optimal configuration, reducing the analysis set from 216 to 27 through an efficient experimental design approach. The results identified the optimal structure as having an aspect ratio of 3.333, four loops, a spiral radius of 6–7 mm, a flow rate of 3 mL/min, a surface roughness of 1 μm, and a particle diameter of 24 μm. Among the evaluated parameters, aspect ratio (61.2%) had the most significant impact, followed by the number of loops (13.9%) and flow rate (9.4%). The optimized design demonstrated high separation efficiency and purity, achieving 97.5% and 97.6%, respectively. The fabrication process involved 3D-printing the channel mold, followed by polydimethylsiloxane (PDMS) casting, validating the durability and scalability of the proposed design. This study integrates simulation and experimental results, providing a robust framework for developing next-generation microfluidic devices and advancing diagnostic and targeted therapeutic applications.

## 1. Introduction

Inertial microfluidics has been extensively studied, leading to the development of numerous applications, primarily for the separation of particles or cells. Physical characteristics play a critical role in the separation of inertial particles in microfluidics. Consequently, refining the architecture of microfluidic devices is a key strategy for improving their efficacy and has garnered significant research interest. To date, numerical simulation-enhanced microfluidic technology has demonstrated considerable potential in microfluidics, single-cell analysis, and early disease diagnosis. However, discrepancies between numerical simulations and clinical or experimental applications remain a challenge. Simulation data have provided deeper insights into the separation process, particularly regarding particle migration in channels with varying contraction-expansion configurations. These findings offer valuable guidance for the design of future particle or cell detection chips [1].

Recent advancements in inertial microfluidics have demonstrated enhanced capabilities in particle and cell separation, particularly for biomedical applications such as cancer diagnostics and circulating tumor cell (CTC) enrichment [1].

In biomedical diagnostics, the isolation of specific cells from tissues or body fluids is crucial for precise detection and analysis, particularly when identifying rare cell populations such as circulating tumor cells (CTCs) [2]. These cells serve as key biomarkers in cancer diagnostics, offering critical insights into tumor metastasis and playing a vital role in diagnosis, staging, treatment planning, and recovery monitoring [3,4]. However, detecting CTCs remains challenging due to their extremely low concentration in blood, typically ranging from 1 to 100 cells per milliliter. This rarity necessitates highly efficient and selective isolation methods to ensure reliable single-cell analysis and meaningful biological insights [5].

Microfluidic technologies have demonstrated significant potential in isolating CTCs, offering high separation efficiency with small sample volumes by leveraging cellular traits such as size, deformability, and electrical characteristics [6]. Contemporary microfluidic methodologies include dielectrophoresis (DEP) [7], acoustic sorting [8], magnetic sorting [9], and optical tweezers, all of which employ external forces for precise cell separation [10]. However, these approaches are often complex and may lack sufficient throughput.

Passive methods, such as deterministic lateral displacement (DLD) and pinch flow filtration (PFF), eliminate the need for external forces and rely on simpler architectures for cell sorting. Nevertheless, they are prone to obstructions and often require auxiliary flows [11]. In contrast, inertial microfluidics offers a highly efficient, label-free technique for cell segregation by size, utilizing hydrodynamic forces to achieve high throughput. This method effectively isolates and concentrates CTCs by leveraging inertial lift and secondary flows, such as Dean vortices, without the need for external forces [12].

Spiral microchannels have gained popularity due to their ability to generate secondary flows that aid in particle separation, as reviewed by Xu et al. (2021) [13]. They are widely utilized in inertial microfluidic applications due to their compact structure and effectiveness in facilitating high-throughput cell sorting [14]. While most research has focused on planar spirals due to their simpler fabrication, three-dimensional spiral channels hold considerable potential for improving circulating tumor cell (CTC) separation by leveraging secondary flows generated by channel curvature and structural distortion [15]. The distinctive flow dynamics of three-dimensional spirals may enhance separation efficiency; however, manufacturing challenges have limited their widespread investigation.

Alternative topologies, such as single-inlet and cascade spirals, offer high purity without the need for sheath flows. Warkiani et al. employed a trapezoidal cross-section to achieve a high throughput of approximately 500 mL/min in Chinese Hamster Ovary (CHO) and yeast cell sorting [16], while Millar et al. demonstrated enhanced purity through cascaded rectangular spirals [17].

The chip design employs a spiral channel to isolate circulating tumor cells (CTCs) from red blood cells (RBCs). This approach enhances both throughput and purity while enabling seamless integration with detection systems such as impedance and imaging, making it suitable for real-time diagnostic applications [18,19]. The chip, fabricated from compact PDMS, measures 2 cm by 3 cm and supports multilayer monitoring while occupying minimal space. Experimental results demonstrate the device’s efficacy, achieving separation rates exceeding 80%, purity rates surpassing 90%, and a concentration fold of approximately 20. This system effectively isolates CTCs, such as SW480, A549, and Caki-1, from RBCs at a flow rate of 1.3 mL/min [20].

Advancements in microfluidic technology enable precise manipulation and analysis of fluids at the microscale, facilitating novel applications in biological diagnostics, environmental monitoring, and chemical analysis [21,22]. Microfluidic platforms leverage distinctive fluid dynamics, such as inertial lift, Dean drag, and secondary flows, to achieve high-throughput, label-free separation of cells and particles [23,24,25,26,27]. This capability for effective sorting without external forces allows particles to migrate toward equilibrium positions based on characteristics such as size, shape, and density [28]. Inertial microfluidics, utilizing passive channel geometries, has become a powerful tool in biomedical applications, enabling continuous, real-time monitoring of health indices through precise cell manipulation and separation [29,30,31].

Computational fluid dynamics (CFD), along with advancements in multi-vortex regulation and multi-material bioprinting, has facilitated the development of intricate tissue models and organ-on-chip systems, supporting applications in cancer diagnostics, blood analysis, and environmental assessments [32,33]. These advancements highlight the transformative potential of microfluidics across biological, environmental, and engineering domains, offering scalable and efficient solutions to complex challenges [34].

This study aims to introduce an innovative, integrated approach for CTC enrichment and real-time diagnostic applications by leveraging the adaptability, scalability, and cost-effectiveness of these systems, thereby positioning microfluidics as a powerful tool in the advancement of precision medicine and beyond.

Despite the increasing research on spiral designs, a standardized study assessing the influence of each parameter on the process is still lacking [35]. Previous studies have attempted to isolate various elements through modifications; however, the specific parameters affecting the process and their respective impacts remain unclear.

This study takes a different approach by systematically investigating how high efficiency and purity can be achieved, rather than merely presenting the final values. This is accomplished through a combination of statistical analysis and numerical simulations, followed by experimental validation to ensure the accuracy and reliability of the findings. By integrating these methodologies, this work provides a comprehensive, data-driven optimization framework, offering deeper insights into the fundamental mechanisms underlying efficient particle separation.

This study uniquely combines Taguchi methods with COMSOL (ver.5.4) simulations to significantly reduce computational demands while enhancing design accuracy, making it, to the best of our knowledge, the first to systematically optimize spiral microchannel designs with this level of precision. The simulations conducted in COMSOL Multiphysics were optimized using the Taguchi method, which effectively reduced the analysis set from 216 to 27 by leveraging an optimal design of experiments. Through this optimization, the ideal configuration was identified, featuring a 3.333 aspect ratio, four loops, a spiral radius of 6–7 mm, a flow rate of 3 mL/min, a surface roughness of 1 μm, and a particle diameter of 24 μm. Among these parameters, the aspect ratio (61.2%) emerged as the most influential factor, followed by the number of loops (13.9%) and flow rate (9.4%). The optimized design demonstrated exceptional separation efficiency and purity, achieving 97.5% and 97.6%, respectively. These findings underscore the effectiveness of the proposed methodology in achieving high-performance designs and validate its potential for real-world applications.

## 2. Materials and Methods

### 2.1. Spiral Inertial Microfluidics

Inertial microfluidics represents a promising and adaptable method for size-based cell separation and enrichment, having made significant advancements in recent years through the exploration of novel channel geometries, multiplexing strategies, and integration with complementary technologies [13,36,37,38]. The spiral microchannel is a widely utilized design for inertial sorting, leveraging intrinsic hydrodynamic forces during fluid flow for particle separation. It offers operational simplicity, ease of microfabrication, minimal clogging issues, and scalability for high-volume processing [39].

Inertial microfluidics employs inertial focusing and Dean flow for particle and cell focusing and sorting. In a linear channel, particles and cells concentrated at the equilibrium position experience only inertial lift force, while buoyancy, gravity, and Brownian motion are typically negligible [40]. The interaction between the inertial lift force $\left(F_{L}\right)$, as described in Equation (1), and the Dean drag force $\left(F_{D}\right)$, defined in Equation (2), governs the inertial migration and separation of particles in curved microchannels due to secondary flow at the cross-section.

(1) $$F_{L}=C_{l}\;\cdot \;\rho_{f}\;\cdot \;{\left(1.5\;\cdot \;U\right)}^{2}\;\cdot \;\frac{a^{2}}{{\left(\frac{4\;\cdot \;A}{P}\right)}^{2}}$$

where *$\;C_{l}$* is constant, *$\rho_{f}$* is the fluid density, *U* is the flow rate of fluid flow, *a* is the particle diameter, *A* is the area of the cross-section of the microchannel, and *P* is the perimeter. In spiral channels, in addition to the lift force, Dean forces are also significant. The Dean force causes the particles to secondary flow. The Dean force is defined by:

(2) $$F_{D}=3\;\cdot \;\pi \;\cdot \;\mu \;\cdot \;\left(1.8\right)\;\cdot \;10^{-4}\;\cdot \;(\rho_{f}\;\cdot \;U\;\cdot \;4\;\cdot \;A/\left(P\;\cdot \;\mu^{\prime \prime }\right)\;\cdot \;\sqrt{{\left(\left(4\;\cdot \;A/P\right)/2R\right)}^{1.63}}\;\cdot \;a$$

where *R* is the radius of the spiral curvature.

**Figure 1:**
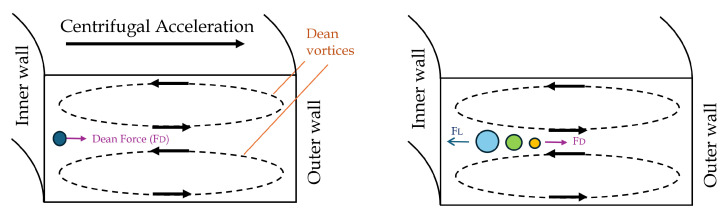
Schematic representation of hydrodynamic forces and secondary flow acting on particles within a spiral microchannel.

Secondary flow in a spiral microfluidic channel refers to the flow components that arise perpendicular to the principal flow direction due to the channel’s curvature and geometry. In these microchannels, secondary flows are induced by centrifugal forces, with their characteristics depending on the channel design, fluid properties, and flow conditions. The forces involved are illustrated in Figure 1.

The parabolic velocity profile of the fluid within the microchannel, characterized by the highest velocity at the center and the lowest velocity near the walls, results in a velocity gradient across the channel’s cross-section. This velocity gradient generates the shear gradient-induced lift force $\left(F_{LS}\right)$, which drives particles away from the microchannel’s central axis. Simultaneously, the interaction of particles with the channel walls produces a wall-induced lift force $\left(F_{LW}\right)$, which pushes particles away from the walls and towards the central region, as depicted in Figure 1. These two forces together constitute the net lift force $\left(F_{L}\right)$, as described in the equation.

In curved microchannels, the combined effects of pressure-driven flow and centrifugal forces give rise to secondary flow patterns known as Dean flows. Dean flows are characterized by two symmetrical counter-rotating vortices located at the upper and lower boundaries of the microchannel’s cross-sectional plane. These flows exert a Dean drag force $\left(F_{D}\right)$ on the particles, directing them towards both the center and the outer wall of the channel, as shown in Figure 1. The intensity of the Dean flow is quantified using the dimensionless Dean number [41], which measures the strength of the secondary flow and is defined in Equation (3).

The Dean number is defined by:

(3) $$De=Re\;\cdot \;D_{H}/\sqrt{D_{H}/2\;\cdot \;R}$$

where $D_{H}$ is the hydraulic diameter.

Microchannel systems are governed by specific equations, as shown below. The ratio of $F_{L}$ to $F_{D}$ is crucial in determining particle positioning. This study aims to quantify the extent to which each parameter influences the system’s efficiency.

Flowing particles may experience additional forces, such as viscous drag, buoyancy, and centrifugal forces; however, these forces are negligible in this context and can be disregarded [42,43].

Particles or cells rapidly migrate to their equilibrium positions due to the combined effects of the Dean drag force and the inertial lift force. Particles of different sizes can be distinguished by their distinct equilibrium locations. The magnitudes of $\left(F_{D}\right)$ and $\left(F_{L}\right)$ depend on the particle size and flow rate. The equilibrium position of a particle is established by the balance between $\left(F_{D}\right)$ and $\left(F_{L}\right)$, meaning that particles of varying sizes will be separated at an optimal flow rate. Given the significant size difference between CTCs and RBCs, they can be effectively separated based on inertial principles [44].

### 2.2. Design

The separation efficiency of the spiral channels was investigated. A spiral structure with a rectangular cross-section was designed using SolidWorks 2020. The spiral design was expanded into nine variations. The loop numbers 2, 3, and 4 refer to the number of complete turns in the spiral configuration. The cross-sectional dimensions of 350×150, 420×120, and 500×100 μm correspond to aspect ratios (w:width/h:height) of 2.333, 3.333, and 5, respectively. The units for cross-sectional measurements are given in μm.

**Figure 2:**
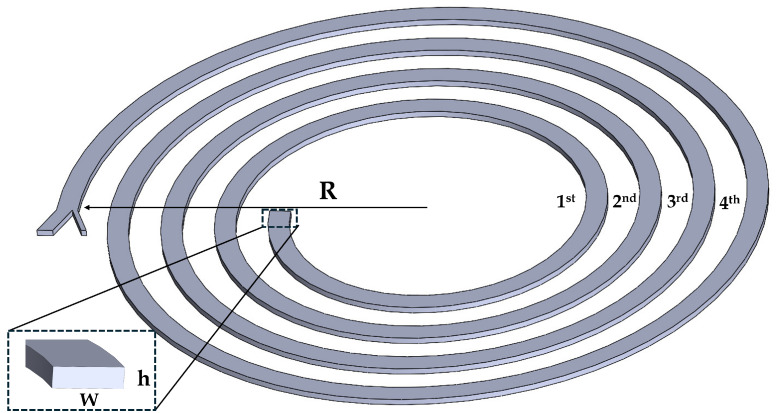
Illustration of spiral design parameters, including channel width (w), height (h), curvature (R), and number of turns, which affect fluid dynamics and particle migration in the microfluidic device microfluidic device.

The spiral channel features three different radius (R) values: 5, 6, and 7 mm. Additionally, the flow speed, roughness, and particle diameter exhibit three variations. The flow rate values are 1.5 mL/min, 2 mL/min, and 3 mL/min. The channel roughness values are 0 (no roughness), 0.5, and 1 μm. The roughness effects were explicitly incorporated into the model by modifying the channel surface and introducing structural features along the inner surface to simulate their impact on flow dynamics and particle migration. The particle sizes considered are 12, 18, and 24 μm. Some of these parameters are illustrated in Figure 2, and all variables are detailed in Table 1.

### 2.3. Statistical Optimization

In this study, the Taguchi method in Minitab software v.2025 (Minitab, State College, PA, USA) was employed as a fractional factorial design to optimize the simulation parameters while minimizing the number of required trials. An L27 orthogonal array was selected, allowing for the analysis of six factors at three levels each, effectively reducing the total number of experiments from 216 ($6^{3}$) full-factorial runs to 27 trials. The Taguchi method in Minitab primarily focuses on determining the main effects of each parameter, providing valuable insights into their influence on separation performance. While some two-factor interactions can be inferred based on the experimental design, higher-order interactions are not explicitly captured due to the fractional factorial nature of the design. This approach ensures an optimal balance between experimental feasibility and statistical robustness, enabling a systematic evaluation of key parameters affecting separation efficiency and purity.

### 2.4. Computational Analysis

The 27 analyses were designed using the Taguchi method in Minitab software, after which the designs were imported into COMSOL Multiphysics for multiphase flow simulations. COMSOL Multiphysics 5.4 finite element software (COMSOL Inc., Stockholm, Sweden) was employed to simulate Newtonian fluid dynamics in the four proposed spiral microchannel topologies. Numerical simulations were conducted within a computational domain consisting of over 45,000 elements, distributed as follows: 17,596 tetrahedral, 27,352 prism, 36 pyramid, 13,716 triangular, and 36 quadrilateral elements. The mesh quality was evaluated based on the skewness criterion, yielding an average element quality of 0.4749. This level of mesh quality ensures a suitable balance between computational load and the resolution necessary for accurately capturing fluid dynamics and particle trajectories within the microfluidic channel. The channels were constructed by importing 3D geometries from Dassault Systèmes SolidWorks (Waltham, MA, USA) and employing mapped meshes, which were iteratively refined to achieve an optimal number of elements. Once the initial conditions (inlet velocity, outlet pressure) and boundary conditions (no-slip at the walls) were appropriately defined, each model was solved using the GMRES (Generalized Minimal Residual) iterative solver to simulate a multiphase, incompressible, laminar flow governed by the Navier–Stokes equations. In this study, one-way coupling (1W) within the Lagrangian Particle Tracking (LPT) module in COMSOL was applied, as the bead volume fraction was maintained below 10%. In this framework, the fluid flow influences particle motion, but the particles do not affect the flow field. Boundary conditions represent a unilateral analysis.

### 2.5. Experiment

#### Chip Preparation

**Figure 3:**
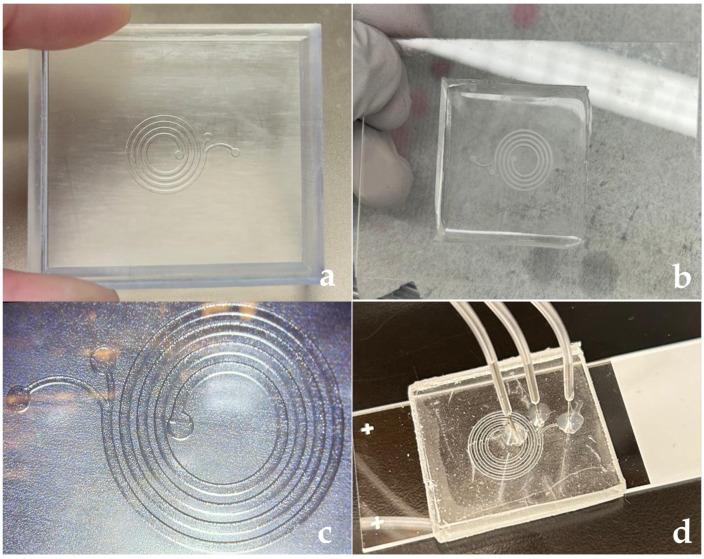
Microfluidic chip fabrication process: (**a**) 3D-printed mold for the chip, (**b**) bonded PDMS and glass assembly, (**c**) microscopy image of the chip, (**d**) attached tubing connections.

The mold for the selected design (High Clear Resin, Anycubic, Shenzhen, China) was precisely fabricated using clear resin with a Digital Light Processing (DLP) 3D-printer (Elegoo, Shenzhen, China) [45]. The entire device was fabricated from PDMS within the mold, while the molds for the spiral channels were produced using various manufacturing techniques. A 10:1 mixture of base and curing agent was poured into the mold for PDMS casting. The mold was then placed in a vacuum-drying oven for one hour to eliminate air bubbles. After degassing, the mold was baked at 37 °C overnight to obtain the cured PDMS replica. Once the PDMS was removed from the mold, a punch was used to create apertures for liquid flow. The replica was bonded to a clean glass slide using a plasma machine (Harrick Plasma, Ithaca, NY, USA) for 75 s at 100 watts. The bonded device was then subjected to a 0.5 kg weight for 5 min to enhance bonding stability. The inlet and outlet needles were inserted and secured using UV light exposure for 10 s. The preparation process is illustrated in Figure 3.

**Figure 4:**
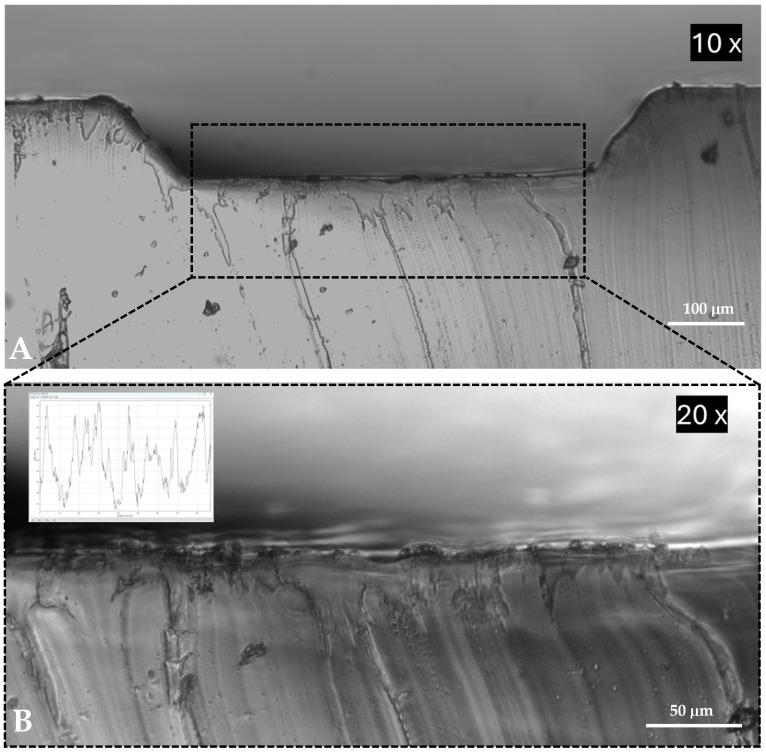
Microscope image showing the surface roughness profile of the (**A**) inner cross-section of the microchannel and (**B**) with a closer view.

To measure the surface roughness, PDMS was cut from the channel cross-section and imaged using a ZEISS microscope, as illustrated in Figure 4. The surface roughness was then analyzed using ImageJ^®^ Fiji v.1.54f (Media Cybernetics, Silver Spring, MD, USA) to calculate the arithmetic average roughness ($R_{a}$) value. The $R_{a}$ value was determined to be 3.71 μm using Equation (4).

(4) $$R_{a}=\frac{1}{L}\int_{0}^{L}\left|y\left(x\right)\right|\;dx$$

where *L* denotes the evaluation length, and y(x) represents the vertical deviation of the surface profile from the mean line at position *x*.

### 2.6. Particle Preparation

Cell separation in inertial microfluidic systems is primarily influenced by size, shape, and density. Circulating tumor cells (CTCs) are generally larger than blood cells, enabling their separation using size-based techniques. In this study, fluorescent polystyrene particles with sizes and densities similar to those of CTCs were used as surrogates. These particles provide well-defined models for size-dependent inertial migration while eliminating biological variability, making them suitable for the initial validation of the system [46].

For the verification experiments involving fluorescent particles, 24 μm (yellow) and 6 μm (Nile Red) fluorescent polystyrene microspheres (Spheretech Inc., Lake Forest, IL, USA) were utilized. The calculated density of the polystyrene particles was 1.05 g/cm^3^, resulting in a concentration of $2.18\times 10^{6}$ particles/mL for 24 μm particles and $1.17\times 10^{7}$ particles/mL for 6 μm particles.

To achieve optimal particle suspension, 20 μm of particles was diluted in 1 mL of PBS (phosphate-buffered saline containing 0.1% Polysorbate 20 to inhibit particle aggregation) in a microcentrifuge tube [47]. After vortexing, the large yellow particles exhibited compatibility with EGFP, whereas the small Nile Red particles were compatible with Cy3 under a fluorescent microscope (ZEISS, Oberkochen, Germany).

#### Preparation of the Experimental Setup

The syringe pump (Braintree Scientific Inc., Braintree, MA, USA) was configured to deliver fluid at a rate of 3 mL/min, with a syringe diameter of 13.5 mm. The syringe was attached to the intake, while two outlets were connected to separate reservoirs, designated as the inner outlet and the outer outlet.

**Figure 5:**
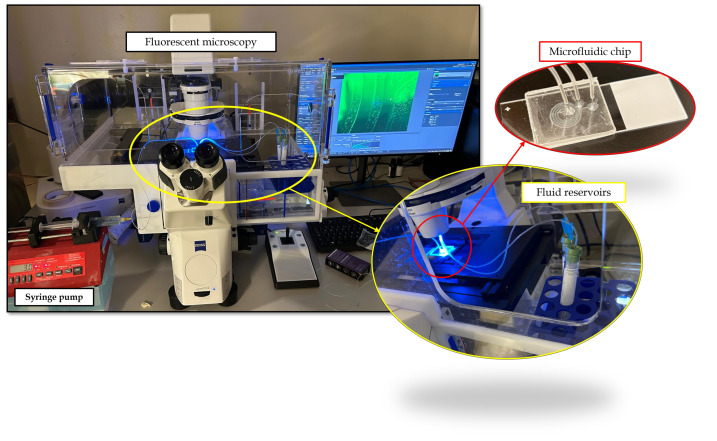
Experimental setup: Fluid is delivered by a syringe pump, flows through the microchannels, and is collected in designated reservoirs for analysis under a fluorescent microscope.

In each experiment, the sample was introduced into the microfluidic device at a specified flow rate via a syringe pump to ensure stable and continuous microflow. A 10 mL syringe was connected to the device using Tygon® flexible plastic tubing (Saint-Gobain, Northborough, MA, USA). (internal diameter: 0.5 mm; length: 30 cm), as shown in Figure 5.

Before use, the device system was first rinsed with 70% ethanol, followed by ultra-purified water, and then with a PBS working buffer. An inverted fluorescent microscope equipped with a charge-coupled device (CCD) camera (Axiocam 705 mono, ZEISS, Oberkochen, Germany) was used for imaging. Images and data were processed and analyzed using ImageJ^®^ Fiji.

## 3. Results

### 3.1. Computational Analysis

A rectangular spiral channel was utilized to investigate the optimal separation parameters required to achieve high separation performance.

**Figure 6:**
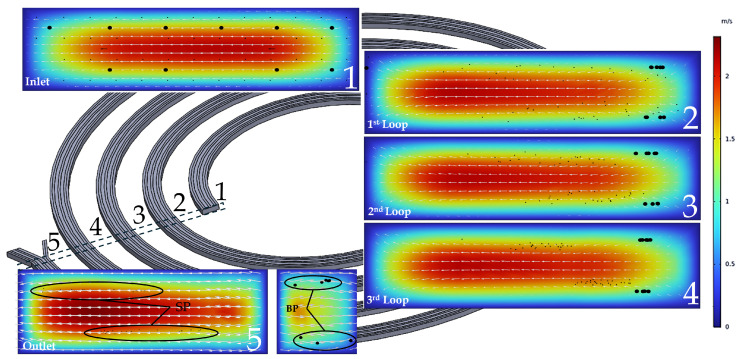
Simulation of particle separation under two-phase flow conditions: Hydrodynamic forces guide small particles (SP) to the outer outlet and big particles (BP) to the inner outlet.

**Figure 7:**
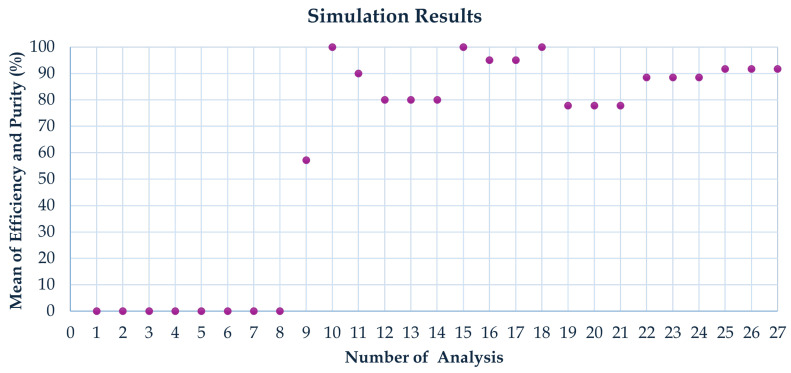
The average efficiency and purity of 27 Comsol Multiphysics simulation results.

The variation in the focal location of 6 μm and 24 μm particles as a function of flow rate in the rectangular channel is illustrated in Figure 6. A flow rate range of 1.5 mL/min to 3 mL/min was analyzed. The 6 μm and 24 μm particles were concentrated at different channel outlets at a flow rate of 3 mL/min. This result was derived from 27 computational simulations, as shown in Figure 7. Numerical annotations have been incorporated into the graph to indicate the degree of result overlap or proximity. Consequently, it was concluded that the optimal flow rate for separation is 3 mL/min. Additionally, the outcome for a flow rate of 4 mL/min was analyzed; however, separation was not clearly distinguishable at this rate. While separation purity improves as the flow rate increases, separation efficiency decreases with further increases in flow rate.

In the rectangular channel, the flow rate must be close to the crossing point in Figure 6 to achieve high purity and efficiency. Figure 6 illustrates that at a flow rate of 3 mL/min, both separation purity and efficiency reached 100%.

### 3.2. Statistical Optimization

The results, consisting of 27 values from COMSOL Multiphysics simulations, were entered into Minitab for comprehensive statistical analysis. An analysis of variance (ANOVA) was performed to assess the significance of each factor and its influence on overall performance. Main effect and interaction plots were generated to visualize the impact of different parameters and their combinations on the outcomes.

**Figure 8:**
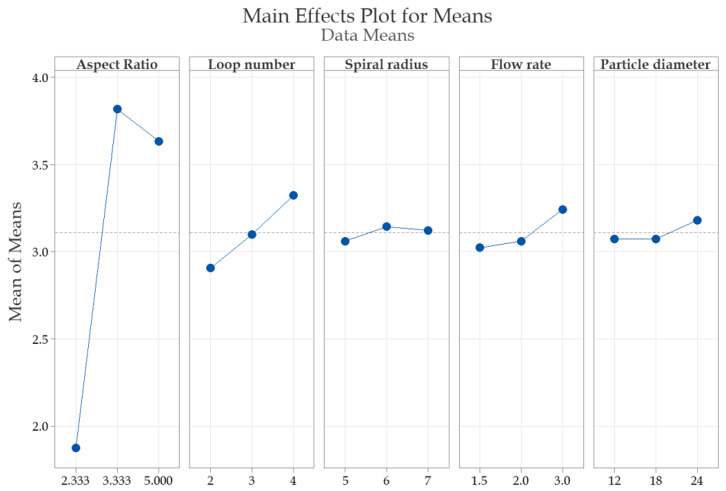
Graphical analysis of the effects of experimental factors on the signal-to-noise ratio (SN Ratio): Higher SN Ratio values indicate improved performance of the response variable.

Following the statistical analysis conducted using Minitab and the implementation of the Taguchi method, as illustrated in Figure 8, the software identified the most effective parameters for optimizing microfluidic devices. The data indicate that the signal-to-noise (SN) ratios increase significantly with a higher aspect ratio, which aligns with the response table. The loop number and spiral radius factors exhibit a positive correlation, with SN ratios increasing as these values rise; however, their impact is less pronounced than that of the aspect ratio. Additionally, flow rate and surface roughness positively influence the enhancement of these parameters, though their contributions to signal-to-noise ratios are relatively minor. The particle diameter demonstrates a negligible impact on SN ratios, suggesting that it may play a less significant role in optimizing the design for separation efficiency.

The signal-to-noise (SN) ratios were computed to assess the robustness of each design against uncontrollable variables, and the optimal combination of factor values was identified accordingly.

The response table (Table 2) for the signal-to-noise ratio indicates that, based on the Delta and Rank columns, aspect ratio (61.2%) was the most influential factor, followed by loop number (13.9%), flow rate (9.4%), spiral radius (5.7%), roughness (5.4%), and particle diameter (4.4%). This response table provides a clear understanding of the relative impact of each parameter, which may be valuable for optimizing microfluidic chip design. Emphasizing the significance of the aspect ratio is particularly useful in refining design recommendations, as it plays a dominant role in separation performance.

The model summary table (Table 3) shows that the Standard Deviation of Residuals (S) was 0.255288, suggesting that the model’s predictions closely aligned with the actual data points. The R-squared (R^2^) value indicates that 96% of the variation in the response variable was explained by the model, demonstrating a strong fit. The adjusted R-squared value was 92.58%, confirming that the model performed effectively with the included predictors. The predicted R-squared value was determined to be 85.13%, indicating the model’s efficacy in forecasting new observations. While this value is slightly lower than the adjusted R^2^, it remains robust. This suggests that the model may exhibit a marginal decrease in performance when applied to novel data; however, it maintains overall reliability.

### 3.3. Experimental Validation of the Model

When the results from the COMSOL Multiphysics software were entered into Minitab, the software suggested design parameters that could maximize efficiency and purity. The microfluidic chip fabricated with the recommended parameters was experimentally validated.

Fluorescent polystyrene particles of varying sizes were used as verification samples. Calculating separation purity and efficiency required measuring the quantity of target and non-target samples in the solution at the collection outlet, as well as the number of target samples in an equivalent volume of solution at the waste outlet. Consequently, the hemocytometer technique was employed to enumerate particles, using Zeiss fluorescent microscopy to capture images. Nine tests were conducted with different chip samples, and the images were analyzed with ImageJ^®^ Fiji to determine the mean value, with the standard deviation representing the error.

**Figure 9:**
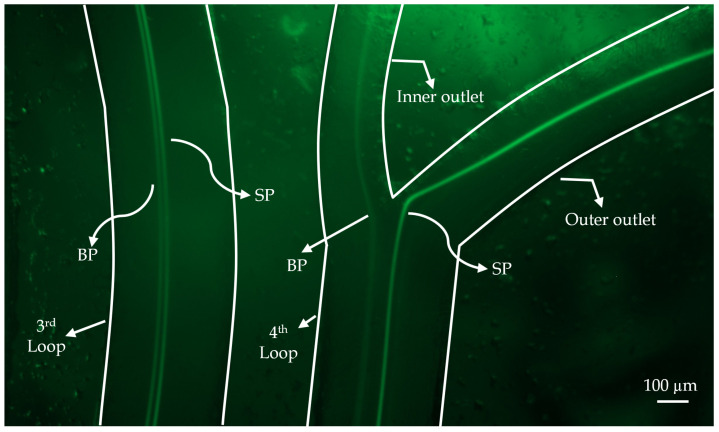
Fluorescence microscope image showing the separation of small particles (SP) and big particles (BP) at the bifurcation point of the microfluidic channel.

**Figure 10:**
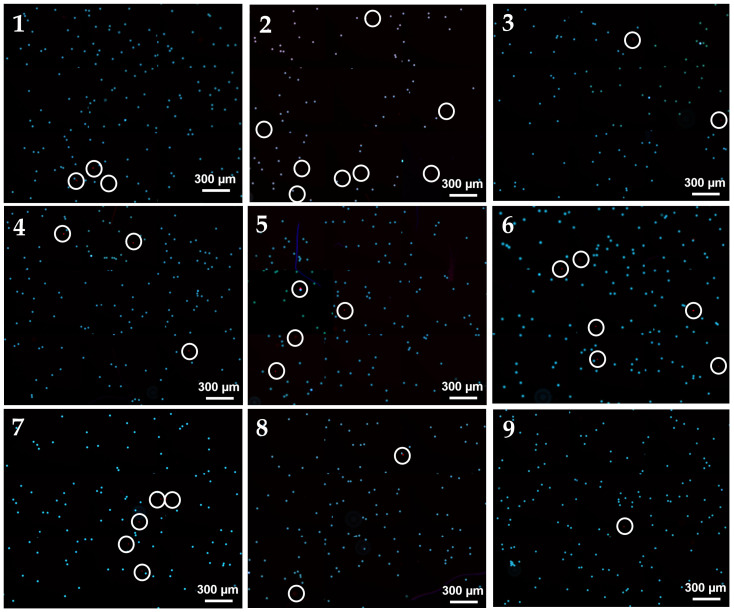
Fluorescence microscope images from the inner outlet showing results from 9 independent experiments (**1–9**) conducted under identical conditions to ensure reproducibility. Big particles are represented in light blue, while small particles are shown in red. The separation of small red particles is highlighted with a circle for illustrative purposes.

The spiral channel with a rectangular cross-section effectively segregated fluorescent particles of different sizes according to their distinct equilibrium positions. The separation patterns of particle focusing and sorting in the spiral were observed via fluorescence microscopy. Figure 9 illustrates the fluorescence trajectory images near the outflow bifurcation. As demonstrated in the COMSOL analysis (Figure 6), successful separation was achieved. Larger particles exited through the inner outlet, while smaller particles exited through the outer outlet. This was confirmed by the samples collected from the reservoirs. To account for potential environmental errors, the experiment was repeated nine times, and the average efficiency and purity results (Figure 10) were compared with the Minitab-designed parameters, which indicated 100% efficiency. An optical microscope captured the sorting processes at the channel outlets, with the flow driven by a syringe pump supplying the input material.

The experimental results may occasionally deviate from the initial design. Contaminants in the fluid, temperature fluctuations, non-uniform surface roughness of the channel’s inner walls, and slight variations in particle sizes during the experiment may have contributed to discrepancies between the experimental results and the simulations. Additionally, separation purity and efficiency were evaluated and calculated from the sample and waste solutions collected from the channel outputs to ensure accurate performance assessment. The separation performance of the proposed chip was assessed using the metrics of throughput, purity, and efficiency. Separation throughput refers to the sample processing velocity of a microfluidic chip, primarily determined by the inlet flow rate or sample processing speed. Separation efficiency indicates the system’s ability to isolate the target sample from the mixed solution.

The separation purity (Equation (5)) and separation efficiency (Equation (6)) are defined as follows [48]:

(5) $$Separation\;Purity\left(\%\right)=\frac{\mathrm{Number}\;\mathrm{of}\;\mathrm{target}\;\mathrm{particles}\;\mathrm{at}\;\mathrm{the}\;\mathrm{inner}\;\mathrm{outlet}}{\mathrm{Total}\;\mathrm{number}\;\mathrm{of}\;\mathrm{particles}\;\mathrm{at}\;\mathrm{the}\;\mathrm{inner}\;\mathrm{outlet}}$$

(6) $$Separation\;Efficiency\left(\%\right)=\frac{\mathrm{Number}\;\mathrm{of}\;\mathrm{target}\;\mathrm{particles}\;\mathrm{at}\;\mathrm{the}\;\mathrm{inner}\;\mathrm{outlet}}{\mathrm{Number}\;\mathrm{of}\;\mathrm{target}\;\mathrm{particles}\;\mathrm{at}\;\mathrm{the}\;\mathrm{intake}}$$

The experiments confirmed that the optimal input flow rate for the multistage channel was 3 mL/min. Figure 9 illustrates that a diffuse streamline of 24 μm particles was concentrated near the inner wall of the rectangular spiral channel, whereas the elevated flow rate prevented the clear visualization of 6 μm particle trajectories near the outer wall. As the flow rate decreased, the streamlines of small particles on either side and the streamlines of large particles at the channel center became discernible. The solution was purified following separation and focusing in the first and second phases. Subsequently, to quantify the normalized intensity, an adjusted threshold was applied to Figure 10. The experiments indicated that the separation purity and efficiency were 97.6% and 97.5%, respectively. These high efficiency and purity values demonstrate that the COMSOL and Minitab simulations were conducted with a high degree of accuracy. Consequently, the experimental findings for various particles confirmed that the computational analysis within the microfluidic chip exhibited strong consistency in particle sorting.

Based on Table 4, statistical analysis confirmed that any variation from the expected efficiency was not significant, with a *p*-value of 0.224 (>0.05). The 95% confidence interval [97.09%, 99.00%] further supports the robustness of the separation process, validating the reliability of this study.

## 4. Discussion

Various microfluidic separation techniques utilize external forces to manipulate cells, including dielectrophoresis (DEP), acoustic sorting, magnetic sorting, and optical tweezers. These methods rely on electric fields, ultrasonic waves, magnetic fields, or laser-based trapping, respectively, to achieve precise separation. However, the requirement for specialized equipment and external energy sources increases operational complexity and cost. Additionally, depending on the applied forces, these methods may introduce potential stress or damage to delicate biological cells, particularly when high-intensity fields or prolonged exposure are involved [49]. Recent efforts have introduced hybrid microfluidic designs that integrate centrifugal and magnetic forces to enhance CTC separation efficiency. Such approaches provide a valuable comparison to the inertial-based separation techniques used in this study.

In contrast, the inertial microfluidic approach used in this study operates without external forces, relying solely on hydrodynamic effects such as inertial lift and Dean vortices to achieve size-based separation. This label-free and passive nature offers significant advantages, including reduced cost, ease of implementation, and minimal impact on cell viability [38]. Moreover, inertial microfluidic devices enable high-throughput processing, making them highly scalable for clinical and industrial applications. While maintaining the aspect ratio helps preserve certain flow characteristics, absolute values also influence separation performance. Further studies are needed to systematically analyze how scaling affects efficiency and purity. In this work, the primary objective was to investigate which parameters have the most significant impact on achieving high separation efficiency and purity. Future research will focus on evaluating aspect ratio variations to assess the scalability of the current findings. By eliminating the need for additional force fields, this technique presents a more practical and biocompatible alternative for continuous cell separation applications.

Despite the efficiency of inertial microfluidic platforms, challenges such as channel clogging, throughput limitations, and separation accuracy remain significant obstacles [37]. These limitations must be carefully considered when designing microfluidic devices for real-world applications, particularly in clinical and industrial settings.

The slight discrepancies between computational and experimental results may be attributed to several factors. First, microfabrication tolerances may introduce minor variations in channel geometry, which are not accounted for in idealized simulations [37]. Additionally, wall tapering effects in the fabricated channels may influence flow dynamics and particle focusing, leading to deviations from simulated predictions. Measurement errors, such as microscope resolution limitations and particle tracking inaccuracies, may also contribute to these differences. Furthermore, computational models typically consider individual particle behavior, whereas in experiments, higher particle concentrations can lead to interparticle interactions, altering separation efficiency [16]. To minimize these discrepancies, incorporating manufacturing tolerances into simulations, improving imaging resolution, and conducting tests at varying particle concentrations could provide further insights and refinements.

The results demonstrate the effectiveness of integrating flow dynamics expertise with statistical optimization to address existing gaps in microfluidic device design. This approach not only simplifies the optimization process but also provides a scalable framework for future applications. Similar optimization strategies have been employed in studies such as Liu et al., where numerical and experimental approaches were combined to enhance microfluidic separation efficiency by systematically optimizing flow rates and channel designs [39].

In the last five years, substantial advancements have been made in the development of separation devices, including non-linear channel topologies for processing real particles. This trend aligns with the increasing focus on numerical analyses of particle migration in non-linear channels, marking a phase of significant progress in this field [13,37]. While polystyrene beads were used as surrogates in this study, it is important to consider the differences that real cells introduce into the separation process. Factors such as deformability, density variation, and surface interactions may influence focusing behavior and efficiency. Future studies will focus on experimental validation using cell samples to assess how these factors impact separation performance and to refine the current model accordingly.

The instantaneous capture and identification of microfeatures, such as particles and cells, are essential in biological research. For instance, Huang et al. highlighted the critical role of particle equilibrium positioning in improving separation efficiency and throughput in curved microchannels [36]. Similarly, inertial separation methods for extracting CTCs from whole blood have proven vital for enhancing cancer diagnostics and guiding treatment decisions [38]. Over the past two years, numerical modeling efforts have increasingly focused on investigating the inertial separation of real particles, including CTCs and bacteria [13,28].

Recent studies, such as [50], have demonstrated that increasing the curvature radius and incorporating straight sections in curved channels enhance separation efficiency. Our results align with these findings, as we also observe that the spiral radius plays a significant role in particle focusing. However, our study extends this approach by utilizing a multi-variable optimization method (Taguchi approach) to systematically refine separation performance.

Despite these advancements, a significant limitation remains: isolated cells often require additional handling processes for identification at the outlet, thereby increasing complexity and elevating contamination risks. To address these challenges, 3D microfluidic chips with varied channel widths and lengths have been developed to improve CTC separation and detection. Omrani et al. demonstrated the enhanced capability of 3D spiral microchannels in overcoming these challenges, showing higher purity levels and reduced contamination risks [15].

This work evaluates the importance of channel dimensions, length, flow rate, and surface properties using computational and experimental methods. The findings extend prior advancements by examining the influence of diverse channel designs on particle migration and separation efficiency in spiral channels with rectangular cross-sections. The complexity of inertial microfluidics in these configurations suggests that the equilibrium position of particles is profoundly influenced by the channel’s cross-sectional aspect ratio, consistent with observations by Russom et al. [41]. The primary force affecting particles is the inertial lift force $F_{L}$, determined by factors such as particle size *a*, cross-sectional aspect ratio AR, Reynolds number Re, and the particle’s position within the channel. Numerical simulations validated through experimental approaches, as recommended by Xiang et al., enhance the reliability of predictive models [37].

Our findings demonstrate that increasing the aspect ratio and loop number significantly enhances separation efficiency, while particle diameter has a reduced effect, as shown in Figure 8. These results align with Zhou et al., who also observed that the aspect ratio strongly influences separation efficiency in curved microchannels [38]. The optimization of parameters such as the aspect ratio, loop number, and flow rate markedly improves device performance. A comprehensive analysis of the impact of each parameter on the signal-to-noise (*SN*) ratio, similar to the approach used by Han et al. [48], provides valuable insights for improving microfluidic methodologies.

Performance consistency and reliability were evident when comparing computational and experimental evaluations. The computational model, with a standard deviation of 0.255288, exhibited minimal variability under optimal conditions, indicating substantial consistency due to controlled parameters. Conversely, the experimental data had a standard deviation of 1.78 across nine observations, potentially attributed to manufacturing conditions, experimental settings, and unobservable errors. These findings are consistent with those of Warkiani et al., who also reported minor experimental deviations when validating computational models [16]. Wall tapering, resulting from the PDMS molding and bonding process, was not explicitly accounted for in the simulations due to the lack of direct experimental measurements. However, since microchannel dimensions are small, even slight variations in sidewall angles could impact fluid dynamics and particle trajectories. In future studies, quantitative evaluation of wall tapering effects and their influence on separation performance should be explored.

The slight difference between computational and experimental standard deviations suggests that while experimental variability is expected, the computational model accurately reflects real-world performance. Enhancing the computational model to address minor inconsistencies observed in experimental settings could further improve predictive accuracy. This comparative analysis underscores the importance of experimental validation in microfluidics research, confirming the model’s applicability and identifying areas for refinement, as similarly highlighted by Millar et al. [17].

In high-concentration suspensions, particle–particle interactions and shear-induced diffusion can alter equilibrium positions, affecting separation efficiency. While this study focused on low-volume fractions where one-way coupling is valid, future work will explore higher concentrations by incorporating interparticle interactions into simulations and experimental validations to assess their impact on inertial focusing.

## 5. Conclusions

This study provides a detailed examination of spiral microchannels designed for efficient particle separation using inertial microfluidics. By systematically analyzing critical parameters, such as the aspect ratio, loop number, flow rate, channel roughness, and particle size, the research identifies their individual contributions to separation performance. Utilizing the Taguchi method, the study successfully reduced the computational burden while maintaining a high level of accuracy in determining the optimal configuration. The results revealed that a channel design with an aspect ratio of 3.333, four loops, a flow rate of 3 mL/min, and a particle size of 24 μm achieved exceptional separation efficiency and purity, reaching 97.5% and 97.6%, respectively. This study addresses one of the major challenges in inertial microfluidics—separation accuracy—by introducing an optimized microchannel design, contributing to more reliable and efficient particle separation [37].

The findings highlight the scalability and versatility of spiral microchannels for high-throughput particle separation, making them promising candidates for applications in diagnostics, biomedical research, and industrial processes. By combining computational optimization with experimental validation, this study offers a robust framework for designing and developing advanced microfluidic devices.

However, minor discrepancies between computational and experimental results were observed, which may stem from fabrication imperfections or experimental variability. Addressing these issues in future studies could further enhance the reliability and precision of the proposed designs. Additionally, exploring alternative geometries, multi-stage channel configurations, or real-time monitoring capabilities could broaden the scope and applicability of these microfluidic systems.

In summary, this work provides valuable insights into the design and optimization of spiral microchannels, reinforcing their potential for next-generation particle separation technologies and advancing the field of inertial microfluidics.

## Figures and Tables

**Table 1:** Design parameters and their variations for analysis.

Aspect Ratio	Level	Value
Aspect ratio	3	2.33–3.33–5.0
Loop number	3	2–3–4
Spiral radius	3	5 mm–6 mm–7 mm
Flow rate	3	1.5 mL/min–2 mL/min–3 mL/min
Roughness	3	0–0.5 μm–1.0 μm
Particle diameter	3	12 μm–18 μm–24 μm

**Table 2:** Response table for signal-to-noise ratios (SNRs).

Level	AR	LN	SR	FR	Rz	PD
1	5.285	8.634	9.008	8.918	8.918	9.212
2	11.622	9.391	9.492	9.289	9.173	9.228
3	11.195	10.077	9.601	9.894	9.731	9.662
Delta	6.338	1.442	0.593	0.976	0.557	0.450
Rank	1	2	4	3	5	6

AR: aspect ratio, LN: loop number, SR: spiral radius, FR: flow rate, Rz: roughness, PD: particle diameter.

**Table 3:** Model summary.

S	R-sq	R-sq (adj)	R-sq (pred)
0.255288	96%	92.58%	85.13%

**Table 4:** Quantification of small beads in the large bead outlet.

Experiment No	Number of Particles at Inner Outlet		Number of Particles at Outer Outlet	
	**Number of Small Particles**	**Number of Big Particles**	**Number of Small Particles**	**Number of Big Particles**
1	3	104	1130	1
2	8	147	1167	5
3	2	166	1228	6
4	3	139	387	1
5	4	144	1142	3
6	6	134	886	1
7	5	104	783	2
8	2	79	856	3
9	1	123	764	1

## Data Availability

Data are available from the authors on request.
